# *De Novo* Generation of Infectious Prions *In Vitro* Produces a New Disease Phenotype

**DOI:** 10.1371/journal.ppat.1000421

**Published:** 2009-05-15

**Authors:** Marcelo A. Barria, Abhisek Mukherjee, Dennisse Gonzalez-Romero, Rodrigo Morales, Claudio Soto

**Affiliations:** 1 George and Cynthia Mitchell Center for Neurodegenerative Diseases, University of Texas Medical Branch, Galveston, Texas, United States of America; 2 Department of Neurology, University of Texas Houston Medical School, Houston, Texas, United States of America; 3 Facultad de Ciencias, University of Chile, Santiago, Chile; University of Alberta, Canada

## Abstract

Prions are the proteinaceous infectious agents responsible for Transmissible Spongiform Encephalopathies. Compelling evidence supports the hypothesis that prions are composed exclusively of a misfolded version of the prion protein (PrP^Sc^) that replicates in the body in the absence of nucleic acids by inducing the misfolding of the cellular prion protein (PrP^C^). The most common form of human prion disease is sporadic, which appears to have its origin in a low frequency event of spontaneous misfolding to generate the first PrP^Sc^ particle that then propagates as in the infectious form of the disease. The main goal of this study was to mimic an early event in the etiology of sporadic disease by attempting *de novo* generation of infectious PrP^Sc^ *in vitro*. For this purpose we analyzed in detail the possibility of spontaneous generation of PrP^Sc^ by the protein misfolding cyclic amplification (PMCA) procedure. Under standard PMCA conditions, and taking precautions to avoid cross-contamination, *de novo* generation of PrP^Sc^ was never observed, supporting the use of the technology for diagnostic applications. However, we report that PMCA can be modified to generate PrP^Sc^ in the absence of pre-existing PrP^Sc^ in different animal species at a low and variable rate. *De novo* generated PrP^Sc^ was infectious when inoculated into wild type hamsters, producing a new disease phenotype with unique clinical, neuropathological and biochemical features. Our results represent additional evidence in support of the prion hypothesis and provide a simple model to study the mechanism of sporadic prion disease. The findings also suggest that prion diversity is not restricted to those currently known, and that likely new forms of infectious protein foldings may be produced, resulting in novel disease phenotypes.

## Author Summary

Prions are the unprecedented infectious agent associated with prion diseases, which are composed exclusively of a misfolded protein. In this study we report the *de novo* formation of infectious prion protein *in vitro*, which when inoculated into wild type animals is able to induce a new disease phenotype with unique clinical, neuropathological and biochemical characteristics. The findings described in this article are very important, because they support the prion hypothesis and provide a simple model for studying the mechanism responsible for the appearance of sporadic prion disease. The data also suggest that the universe of possible prions is not restricted to those currently known, but that likely many new forms of infectious protein foldings may be produced. This is worrisome, because it raises the possibility that novel and perhaps more aggressive infectious prion foldings may spontaneously originate in diverse species, leading to the emergence of new and unpredictable forms of transmissible diseases.

## Introduction

Prions are the proteinaceous infectious agents responsible for Transmissible Spongiform Encephalopathies (TSEs), a group of fatal neurodegenerative disorders, including Creutzfeldt-Jakob disease (CJD) in humans, scrapie in sheep, bovine spongiform encephalopathy in cattle and chronic wasting disease in deer [1]. Compelling evidence support the hypothesis that the infectious agent is composed exclusively by a misfolded version of the prion protein (PrP^Sc^) that replicates in the body in the absence of nucleic acids by inducing the misfolding of the cellular prion protein (PrP^C^) [2]. Recent studies have reported *in vitro* generation of infectious material by inducing or amplifying PrP misfolding, providing strong support for the prion hypothesis [3]–[5].

In addition of the infectious origin, TSEs can be inherited or appear sporadically. The latter is the most common origin in humans, in which there are no known infectious source and no evidence of the disease in the prior or subsequent generations of the patient's family. Sporadic CJD occurs worldwide with an approximate frequency of 1 new case per million people each year [1]. Molecularly, it is thought that sporadic TSEs arise from the low frequency, spontaneous misfolding of the prion protein which then propagates to other PrP^C^ molecules in a manner similar as in the infectious cases.

Based on the knowledge of the molecular mechanism of prion conversion, we have developed the PMCA (Protein Misfolding Cyclic Amplification) technology, designed to mimic PrP^Sc^ autocatalytic replication *in vitro* [6]. In a cyclic manner, minute quantities of PrP^Sc^ (as little as one single particle) induce misfolding of large amounts of PrP^C^ in a process catalyzed by ultrasound waves to multiply the number of converting units. We and others demonstrated that PrP^Sc^ can be maintained replicating *in vitro* indefinitively [3],[7]. More importantly, inoculation of in vitro generated PrP^Sc^ produced disease in wild type animals [3]. The newly generated protein exhibits the same biochemical, biological, and structural properties as brain-derived PrP^Sc^ and maintains the species-barrier and strain-specific characteristics [8]–[10]. These findings represent one of the strongest evidences in favor of the prion hypothesis. However, since infectious material was generated using crude brain homogenates as substrate and starting from small amounts of brain derived infectivity, we cannot absolutely rule out that another agent besides of PrP^Sc^ was replicated during the PMCA process. In this sense a great advance was reported by Supattapone and co-workers with the *in vitro* generation of PrP^Sc^ and infectivity by PMCA using purified PrP^C^ and PrP^Sc^ with the sole addition of a synthetic polyanion [4]. Moreover, these authors described that misfolded and infectious protein was generated *de novo*, in hamsters under conditions in which the possibility of cross-contamination was greatly reduced. The result was a hamster strain very similar to the commonly used 263K hamster strain [4]. Spontaneous generation of PrP^Sc^ and infectivity was also described in an experiment in which a recombinant mouse PrP fragment (residues 89–230) assembled into amyloid fibrils was found to induce a TSE-like disease with PrP^Sc^ formation when injected in transgenic mice overexpressing the same PrP sequence [5]. Although these findings are very interesting, the fact that the disease was transmitted in a first passage only to transgenic animals largely over-expressing the PrP gene and not to wild-type animals is a matter of concern. It is well known that this type of animals develop spontaneously a prion-like disease at advanced age [11]–[13]. In other words, the effect seen might just be an acceleration of the disease process that was set to occur spontaneously at a later time [14].

The main goal of the current study was to identify conditions which permit the *de novo* generation of PrP and infectivity using PMCA in brain homogenates. Our hypothesis was that if sporadic prion disease originates from a low frequency process of spontaneous PrP^C^ misfolding, we should be able to pick up and propagate this event using PMCA. However, in our experience thousands PMCA experiments have only rarely shown PrP^Sc^ formation in control samples without infectious material and in these extremely uncommon cases we could not rule out the possibility of cross-contamination.

## Results

**Figure 1:**
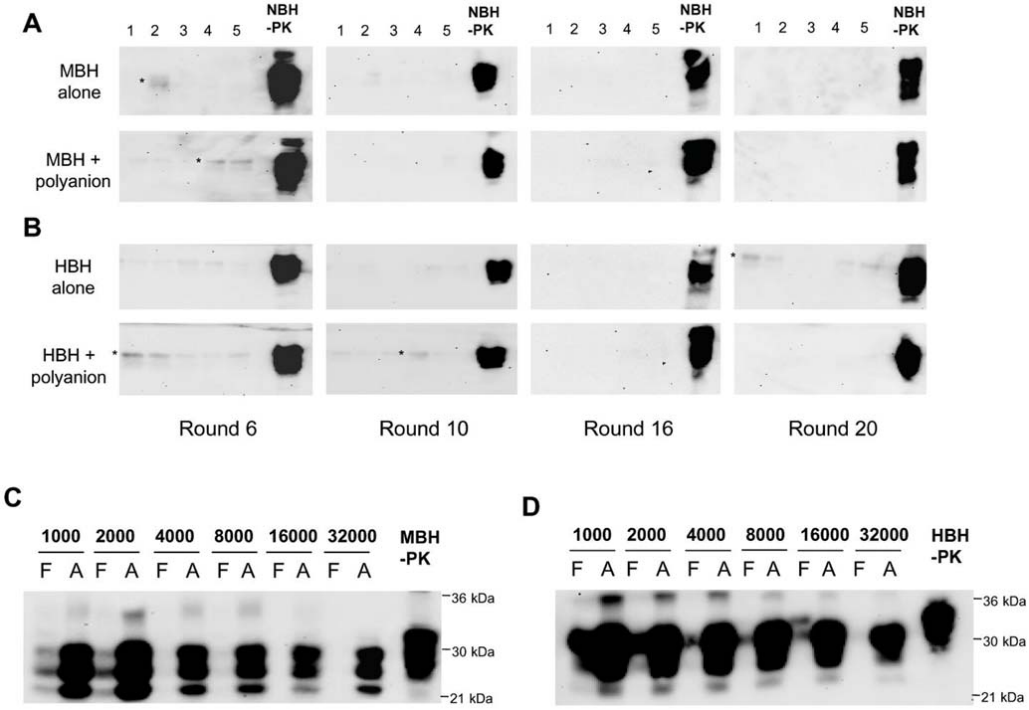
Standard PMCA does not lead to spontaneous generation of PrP^Sc^. Ten different mouse (A) and hamster (B) brain samples were subjected to serial rounds of PMCA in the absence of PrP^Sc^ inoculum. A total of 20 rounds of 144 PMCA cycles were done and after each round the appearance of PrP^res^ after PK digestion was tested by western blot. The figure shows the results obtained in passages 6, 10, 16 and 20, and 5 representative samples of the 10 analyzed. In half of the samples, the PMCA reaction was done in the absence and the other half in the presence of 20 µg/ml synthetic poly-A oligonucleotide. These studies were done in a prion-free room using all new equipment and reagents. In some lanes (labeled with an asterisk) it is possible to appreciate a very faint band with the same molecular weight as PrP^C^, which is the result of incomplete digestion with PK. The efficiency of PMCA using these conditions was tested by running standard PMCA experiments using different dilutions of infected mouse (C) or hamster (D) brain homogenate. The experiment was done using 144 PMCA cycles and the results showed a robust amplification of the signal as previously described [21],[34]. All samples were digested with PK before western blot, except in the normal brain homogenate (NBH), used as control of PrP^C^ migration. MBH: healthy mouse brain homogenate; HBH: healthy hamster brain homogenate; F: frozen samples; A: amplified samples.

To assess more systematically the putative *de novo* formation of PrP^Sc^ *in vitro*, we performed 20 serial rounds of PMCA using 10 different mouse and hamster uninfected brains. For this experiment we used the standard PMCA conditions, in which each round consists of 144 cycles of 30 minutes incubation followed by a 20 s pulse of sonication. To minimize the possibility of cross-contamination, the experiment was done in a room that has never been exposed to prions and using all new equipment and reagents. Half of the samples were also incubated in the presence of poly-A oligonucleotide, a synthetic polyanion which have been shown to promote PrP^Sc^ formation *in vitro* by PMCA [4],[15]. The result of this experiment did not show protease-resistant PrP in any of the conditions tested neither in mouse (Fig. 1A) nor in hamster (Fig. 1B) samples. The efficiency of the equipment and reagents used to replicate prions was confirmed thereafter in an experiment in which standard PMCA was done to amplify diverse quantities of brain-derived mouse or hamster PrP^Sc^ (Fig. 1C and D). These results indicate that under standard PMCA conditions in which cross-contamination has been avoided, spontaneous formation of hamster or mouse PrP^Sc^ does not occur.

**Figure 2:**
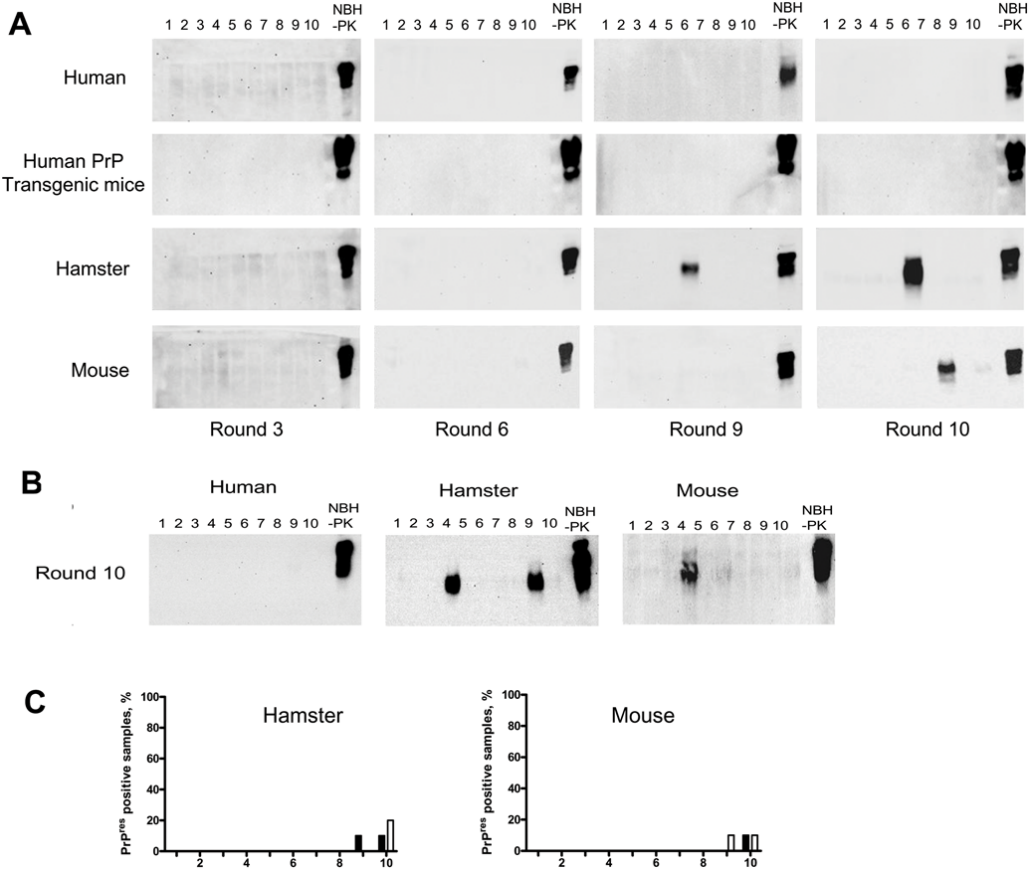
*De novo* generation of PrP^Sc^ in diverse animal species. (A) Samples from 10 different healthy brain homogenates from human, hamster, mouse or humanized transgenic mice were subjected to successive rounds of extended PMCA. Each round consisted of 240 PMCA cycles (30 min incubation followed by a 20 s pulse of sonication). Aliquots of each sample were treated with PK and analyzed by western blot. The results of rounds 3, 6, 9 and 10 are shown. (B) The same experiment as in A was repeated in a prion-free laboratory with all new equipment and reagents to reduce the possibility of cross-contamination. Only the round 10 is shown, but overall the results are very similar as those described in panel A. All samples were digested with PK before Western blot, except in the normal brain homogenate (NBH), used as control of PrP^C^ migration. (C) Rate of spontaneous generation of protease-resistant PrP (PrP^res^) in different species at each of the PMCA rounds performed. Full bars represent the results obtained in the experiment conducted in our standard facility (as shown in panel A) and empty bars the experiment done in prion-free room with all new equipment and reagents (as shown in panel B).

We reasoned that replication starting with a spontaneously formed and unstable intermediate of PrP^Sc^, as the expected precursor of sporadic disease, may require different conditions to propagate than a mature, stable PrP^Sc^ form. Thus, to further attempt *de novo* generation of PrP^Sc^ we tested various changes of the PMCA conditions (see below). In particular we assessed whether longer incubation periods before dilution in a second round may benefit amplification of *de novo* generated prions. We also tested whether human brain material may have a greater propensity to produce PrP^Sc^ spontaneously, because of the known incidence of sporadic disease in the population [16]. After several, extended serial rounds of PMCA, consisting of 240 PMCA cycles (5 days of sonications every 30 min each), we observed a protease-resistant PrP band similar to PrP^Sc^ in hamsters and mice, but not in humans or transgenic mice over-expressing human PrP (Fig. 2). Interestingly, the rate of spontaneous formation as well as the number of PMCA rounds needed to generate *de novo* PrP^Sc^ was different in distinct species. Only 1 out of 10 samples of hamsters and mice gave a PrP^Sc^ signal, which appeared after 9 and 10 rounds of PMCA, respectively (Fig. 2A and C). With human material we never observed spontaneous formation of PrP^Sc^. The variable rate of spontaneous generation of PrP^Sc^ in different species argues against the possibility of cross-contamination as the source of *de novo* PrP^Sc^. To further rule out contamination, the entire experiment was repeated in a prion-free room with all new equipment and reagents. Under these conditions we observed a very similar rate of spontaneous appearance of PrP^Sc^ in different species (Fig. 2B). Again human material did not show signal and hamsters and mice showed low, but detectable levels of PrP^Sc^ spontaneous appearance (20% and 10%, respectively). The PMCA rounds in which the first PrP^res^ signals appeared was also similar in both experiments (Fig. 2C). The efficiency of PrP^C^ from human and humanized transgenic mice brain to undergo conversion into PrP^Sc^ by PMCA was checked by performing a standard seeded PMCA experiment. As shown in Fig. S1, these substrates supported prion replication. At this time we do not know whether PMCA replicated a PrP^Sc^ intermediate already present in the brain homogenate or rather “spontaneous” misfolding was induced during PMCA. In order to gain insight regarding this issue, we subjected the samples of normal brain homogenate to conditions that could increase misfolding, such as heating at 55°C, addition of detergents (SDS or digitonin) and changes in pH (pH 5.0). To attempt stabilizing a pre-formed intermediate, we pre-incubated with shaking the normal brain homogenate for 24 h prior PMCA. None of these conditions resulted in detectable *de novo* formation of PrP^Sc^ in healthy brain homogenate from hamsters (Fig. S2) or human transgenic mice (Fig. S3).

**Figure 3:**
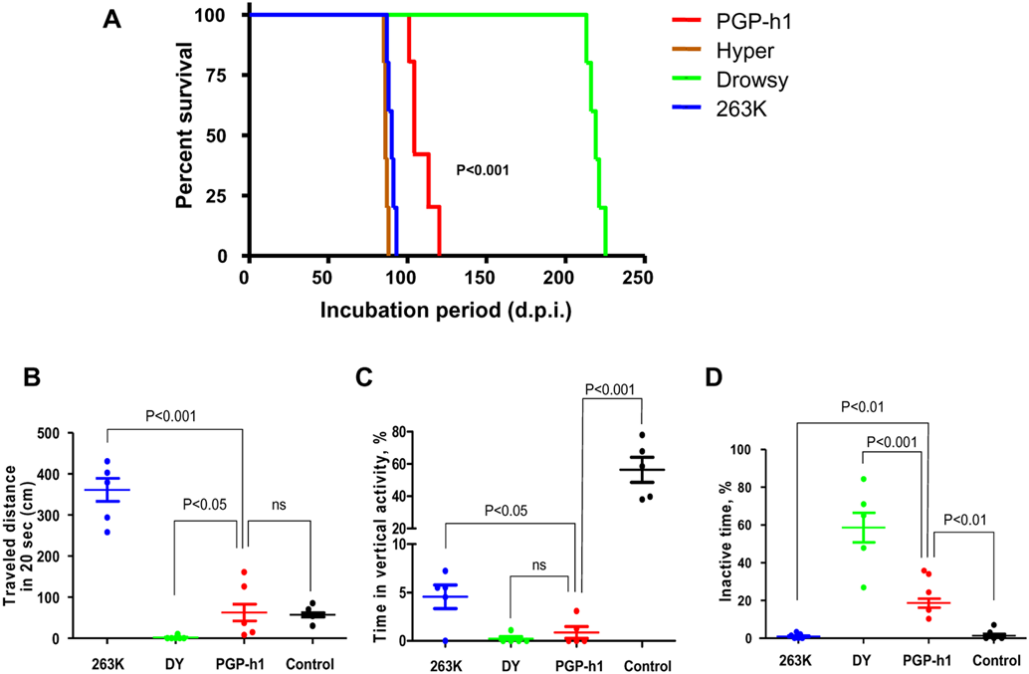
*De novo* generated PrP^Sc^ is infectious, producing a new clinical disease. (A) Groups of 5 hamsters were *intra-cerebrally* (i.c.) inoculated with similar quantities of PrP^Sc^ from either *de novo* generated protein (PGP-h1) or three well-established hamster prion strains, including 263K, HY and DY. Clinical signs were monitored weekly as described in Materials and Methods and when the signs progressed up to the level 4 of our scale, animals were considered sick and sacrificed to avoid excessive pain. This time is recorded as incubation period and is represented in the figure as days post-inoculation (d.p.i.). Differences on the incubation periods produced by the diverse sources of infectious material were analyzed by one-way ANOVA and were found highly significant (P<0.001). The significance of the differences between PGP-h1 and each of the other hamster prion strains were evaluated by the Dunnett Multiple Comparison post test. All differences were statistically significant (P<0.001). Behavioral alterations during the clinical phase of the disease in animals inoculated with various prion strains were assessed by an open field test, as described in Materials and Methods. Hamsters at mid stages of the disease (around 1 week after the first clinical signs were observed) were placed in a corner of the field and its behavior recorded for five minutes. We measured and analyzed total distance traveled during 20 s intervals (B), time that animals spent in vertical activity (C) and inactive time (D). The last two parameters are represented as a percentage of the total time. Tests were done twice for each animal and the result showed in the figure correspond to the average of the two determinations. Behavioral data was analyzed by one-way ANOVA with the Dunnett multiple comparison post-test. Statistical probability that the differences between PGP-h1 and control animals or hamsters inoculated with the other strains are different is indicated in the figure.

To assess whether *de novo* PrP^Sc^ is infectious, we injected *intra-cerebrally* the protein generated using hamster brain homogenate (lane 6 in Fig. 2A) into wild type animals. Simultaneously, we inoculated groups of hamsters with similar quantities of PrP^Sc^ obtained from the brain of animals injected with three well-characterized hamster prion strains: 263K, Hyper (HY) and Drowsy (DY) [17],[18]. All animals inoculated with *de novo* generated PrP^Sc^ showed clear behavioral abnormalities, including tremor of the head, wobbling gait, head bobbing and reduced or absent exploratory behavior, but notoriously we never observed hyperactivity and aggressiveness that are typical of the most common hamster prion strains (such as 263K, Sc237 and HY). The disease progressed quickly, resulting in death between 2–3 weeks after the appearance of the first clinical signs. The average incubation period for the animals inoculated with *de novo* produced PrP^Sc^, termed PMCA-generated prions in hamsters (PGP-h1), was 112.6±5.2 days, which was statistically significantly different from the incubation times obtained for 263K (89.8±1.1 days), HY (86.5±0.65 days) or DY (218.8±2.1 days) (Fig. 3A). We are currently doing serial passages and end-point titration of this new infectious material to investigate its stability and infectivity titer. To further analyze the characteristics of the disease produced by *in vitro* generated PrP^Sc^ we performed behavioral experiments using an open field test. Three different parameters were measured, including distance traveled in a 20 s period (Fig. 3B), vertical activity (Fig. 3C) and percentage of time in which animals remained inactive (Fig. 3D). Animals inoculated with PGP-h1 showed substantially less traveling activity than the hyperactive 263K sick animals, but significantly higher than the lethargic DY hamsters (Fig. 3B). The distance traveled was not substantially different than control uninoculated hamsters, but the PGP-h1 animals have a substantially reduced vertical activity (Fig. 3C) and a much higher rate of inactive time (Fig. 3D) than healthy hamsters. The disease observed in *de novo* inoculated animals differed from the two hamster strains studied also in the percentage of inactive time, which was greater than in 263K but lower than in DY (Fig. 3D). The reduced horizontal and vertical activity and increased inactive time in PGP-h1 sick hamsters compared to healthy animals could be interpreted as increased anxiety or fear, but this is unlikely since similar behavior was observed when the experiment was repeated in the home cage. The abnormal behavior is probably reflecting a social withdrawal and lack of exploratory interest. These findings indicate that the clinical characteristics of the disease produced by inoculation of PGP-h1 PrP^Sc^ are different from the symptoms observed in typical hamster strains, which is also evident by simply observing video recording of these animals (see Video S1 and Video S2 in supporting online material).

**Figure 4:**
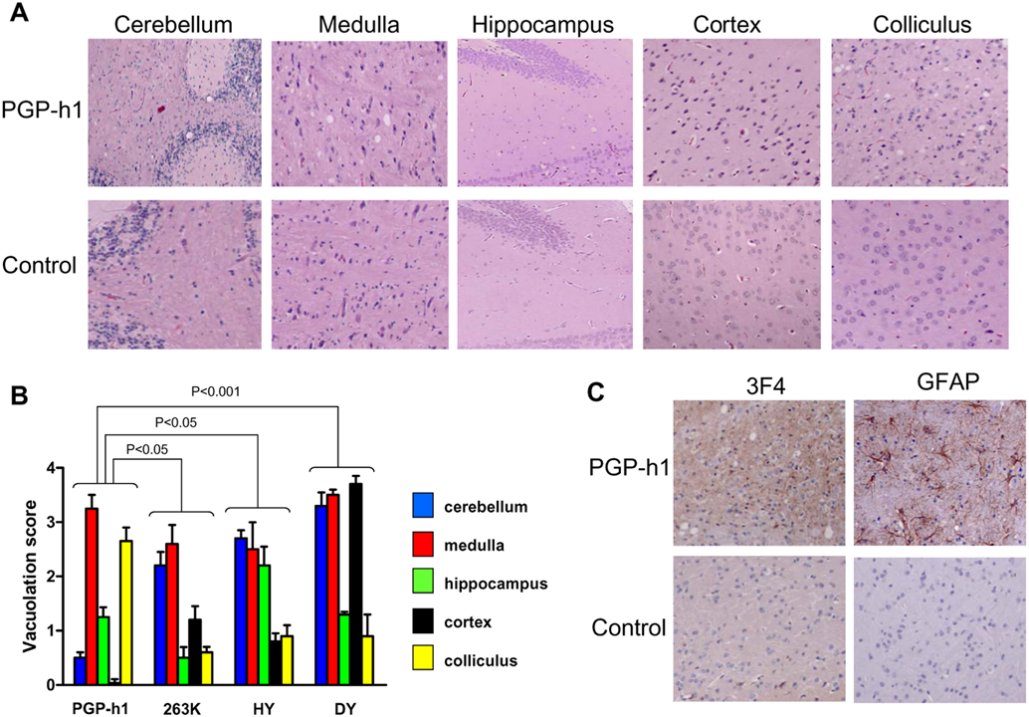
Histopathological brain damage in hamsters inoculated with *de novo* generated prions. (A) Five different brain areas of animals sacrificed with clinical signs of disease induced by PGP-h1 infection and un-inoculated age-matched controls were analyzed histologically for spongiform degeneration after hematoxilin-eosin staining. (B) The vacuolation profile in each brain area was estimated using a semi-quantitative scale, as described in Materials and Methods. We also included in the analysis brain sections from animals inoculated with the other hamster prion strains studied. The values represent the average±standard error of the extent of vacuolation from the 5 animals analyzed in each set. Statistical analysis by two-ways ANOVA, using brain regions and prion origin as the variables indicated that differences were highly significant (P<0.001). To assess the significance of the differences between each known prion strain and PGP-h1, we used the Dunnett multiple comparison post-test and the P values for each combination are shown in the figure. (C) PrP^Sc^ accumulation and astroglyosis were studied by immunohistochemistry using anti-PrP and anti-GFAP antibodies, respectively. In the figure we show the staining in medulla as a representative brain region where substantial damage was observed. Staining of the brain of un-inoculated animals was included as a negative control.

**Figure 5:**
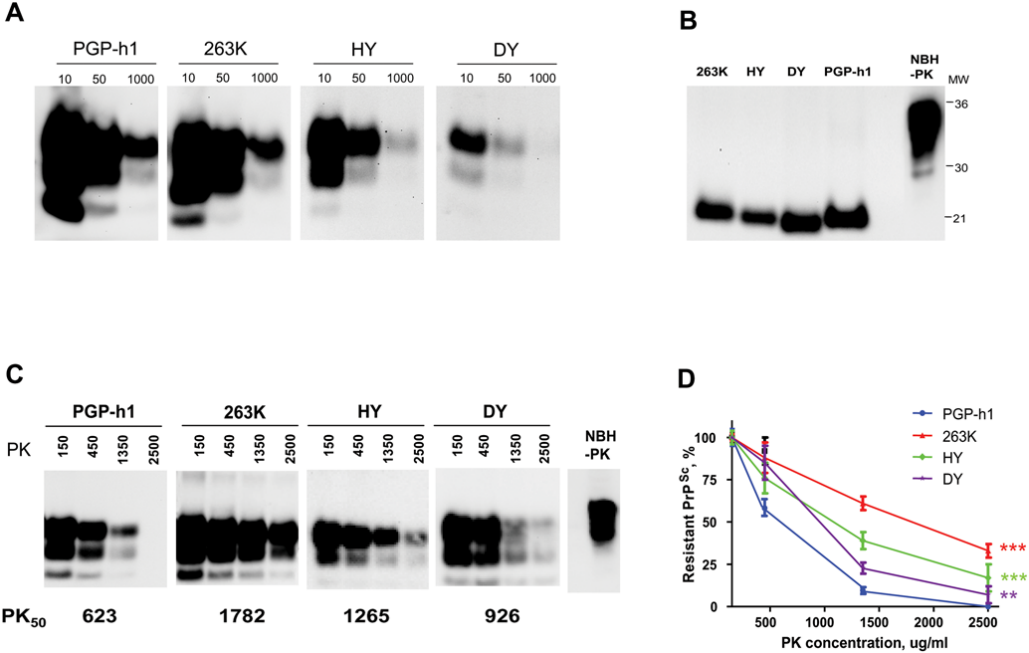
Biochemical characteristics of PrP^Sc^ obtained in animals infected with *de novo* generated prions. (A) To estimate the relative quantity of PrP^Sc^ in the brain of animals inoculated with different prion strains, the tissue was homogenized and aliquots corresponding to a 10-, 50-, and 1000-fold dilutions (respect to the entire brain) were analyzed by western blot after PK treatment. (B) The size of the PK-resistant fragment in each strain was assessed by western blot after treatment with the protease and deglycosylated as described in Materials and Methods. (C) The relative sensitivity of PGP-h1 PrP^Sc^ to proteolytic degradation was studied and compared with that of PrP^Sc^ associated to other hamster strains. For this study, aliquots of brain homogenate were incubated for 60 min at 37°C with the indicated concentrations of PK and PrP^Sc^ signal remaining was detected by Western blot. All samples were digested with PK before Western blot, except in the normal brain homogenate (NBH), used as control of PrP^C^ migration. (D) Densitometric analysis of the western blots of 3 independent experiments as the one shown in the panel C was done to calculate the quantity of PrP^Sc^ digested with each PK concentration. This data enable to determine the susceptibility of PrP^Sc^ from the various sources to PK digestion and to estimate the PK_50_ value, which corresponds to the PK concentration needed to degrade 50% of the protein. The PK_50_ values (expressed in ug/ml of the protease) for each strain are indicated at the bottom of panel C. The data represent the average±standard error. The data was analyzed by two ways ANOVA (with source of the materials and PK concentration as the variables) and the Dunnett multiple comparison post-test. Each set of data was compared to the results obtained with the PGP-h1 strain and significant differences are highlighted with asterisks (_*_ = P<0.05; _**_ = P<0.01; _***_ = P<0.001).

A detailed study of the brain damage in PGP-h1 sick animals showed neuropathological alterations typical of prion disease, including spongiform degeneration, astroglyosis and PrP accumulation (Fig. 4). Extensive vacuolation was observed in medulla and colliculus, whereas the extent of spongiosis was smaller, but detectable, in hippocampus and cerebellum and almost non-existent in cortex (Fig. 4A). Hippocampal spongiosis was mostly detected in areas of the CA1 region rather than in the dentate gyrus layers where vacuoles are frequently observed in other hamster strains. Interestingly, comparison of the vacuolation profile in distinct brain areas with those observed in hamsters affected by other prion strains, showed that the neuropathological pattern of PGP-h1 is clearly and significantly different from all the others (Fig. 4B). The main difference with the previously known strains is the substantial spongiosis in colliculus and the lack of severe damage in cerebellum. PrP^Sc^ diffuse deposits were observed in diverse areas of the brain (Fig. 4C) and a protease-resistant misfolded protein was readily detectable by western blot (Fig. 5A). The quantity of PrP^Sc^ was similar to that observed in 263K infected animals, and was substantially higher than in HY and DY sick hamsters. The glycosylation profile of PGP-h1 PrP^Sc^ is undistinguishable from that of other hamster strains and is dominated by the di-glycosylated band (Fig. 5A). The electrophoretic mobility was assessed by western blot after PK treatment and de-glycosylation with PNGase enzyme. Under these conditions PGP-h1 PrP^Sc^ showed an approximate molecular weight of 21 kDa, not different from 263K or HY PrP^Sc^, but clearly distinct from the 19 kDa obtained with DY PrP^Sc^ (Fig. 5B). Another important biochemical difference among PrP^Sc^ from different strains is the degree of resistance to proteolytic degradation [19]. To measure this, we subjected similar quantities of PrP^Sc^ from diverse strains to increasing PK concentrations. As shown in Fig. 5C, PrP^Sc^ associated with *de novo* generated prions has a significantly lower resistance to proteolysis relative to the misfolded protein from other hamster strains. The PK_50_ value (which represents the protease concentration in which 50% of PrP^Sc^ is eliminated), for PGP-h1 was 623 ug/ml, compared to 1782, 1265 and 926 for 263K, HY and DY PrP^Sc^, respectively (Fig. 5C).

## Discussion

In this study we report spontaneous generation of PrP^Sc^ *in vitro* using a modified PMCA procedure with brain homogenate substrate. Misfolded protein was produced in two different animal species without the need to add seeds of *in vivo* generated PrP^Sc^. At present it is unclear whether PMCA replicated a PrP^Sc^ intermediate already present in the brain homogenate or rather “spontaneous” misfolding was induced by the amplification cycles. In this context, a recent study reported the presence of small quantities of a PrP^Sc^-like protein in healthy brains [20], but it is yet unknown whether this protein is infectious.

Although de novo prion formation was achieved before by PMCA using purified proteins in the presence of synthetic polyanions [4], the purpose of our study was to evaluate whether *de novo* generation of prions is possible under standard PMCA conditions. This is important to assess the validity of using PMCA for prion diagnosis. Another aim was to study whether different species of animals have a distinct propensity to form spontaneous prions under a given set of conditions. Finally, we aimed to characterize the newly generated infectivity and to show that the diversity of prions is perhaps larger than we currently think. Our results indicate that standard PMCA rounds consisting of 144 cycles (or less) did not show spontaneous formation of PrP^Sc^, but longer rounds of 240 cycles were successful in replicating or generating misfolded protein. These findings suggest that *de novo* formation of PrP^Sc^ can be experimentally distinguished from replication of pre-formed PrP^Sc^, indicating that the biochemical, conformational or stability properties of the PrP structures involved in both processes are probably different. Therefore, PMCA can be adapted to produce *de novo* generated PrP^Sc^ and it is likely that more drastic modifications of the procedure may lead to higher rates of spontaneous prion formation. Indeed, the higher rate of *de novo* PrP^Sc^ formation reported in the study by Deleault and colleagues, who used only 48 PMCA cycles per round [4], indicates that using polyanions and purified PrP^C^ may favor spontaneous prion formation, suggesting that some factors in brain homogenate may prevent PrP misfolding.

Our data support the use of PMCA, (under conditions in which PrP^Sc^ *de novo* generation does not occur), for biochemical detection of prions and its potential application for TSE diagnosis. The usefulness of PMCA for highly sensitive and specific detection of prions in biological fluids, has been demonstrated by studies from us and other groups [21]–[25].

Strikingly, the rate and number of PMCA cycles required to produce *de novo* PrP^Sc^ was variable in distinct species. Considering that from the species studied humans are the only one in which sporadic disease is known to occur, we were surprised that *de novo* PrP^Sc^ was never obtained with human brain homogenates under the experimental conditions used. This contrast with the high levels of amplification of human PrP^Sc^ observed in samples seeded with vCJD (Fig. S1) or various sCJD brain homogenates (data not shown). The absence of spontaneous formation of PrP^res^ in humans may be due to the different conditions for the preparation of the tissue, including several hours of post-mortem delay or the lack of perfusion before collection of the brain. However, this is unlikely, because similar results were obtained using brains of transgenic mice expressing human PrP subjected to the same conditions to prepare brain than the other rodent species. Our interpretation of these results is that human PrP^C^ has a lower propensity to initiate misfolding than the rodent protein. The alternative explanation that factors present in the human brain may prevent conversion is unlikely considering the experiments with humanized transgenic mice. The appearance of sporadic disease in humans may simply reflect the longer life span that provides greater chances for stochastic processes of spontaneous misfolding. However, it is also possible that sporadic disease is actually more frequent in animals, because the rate of spontaneous illness in animals has not been systematically studied.

Several lines of evidence enable us to rule out the possibility that *de novo* PrP^Sc^ was the result of cross-contamination. First of all, conventional PMCA that allows us to amplify as little as a single particle of PrP^Sc^ did not show spontaneous formation of misfolded protein, even after extensive PMCA cycling (Fig. 1). Second, the rate of spontaneous generation of prions in different species does not correlate with the availability of infectious material in our lab. Moreover, the variable rate was surprisingly constant among different experiments, arguing that it is dependent on some intrinsic properties of the protein rather than a stochastic event as cross-contamination. Third, an experiment done using strictly prion-free equipment and reagents, performed in a new laboratory that was never exposed to prions and done by a person who has never been in contact with prion material showed very similar results as the one carried out in our standard facilities. Finally, the biochemical, structural and biological properties of *de novo* generated PrP^Sc^ in hamster were substantially different to those of various conventional hamster prion strains. Although all these evidences strongly indicate that the results are not due to cross-contamination, this possibility cannot entirely be ruled out.

Inoculation of wild type hamsters with *de novo* generated PrP^Sc^ produced disease in all animals. Strikingly, many of the disease characteristics were substantially different to those observed with several other hamster prion strains. In this study we directly compared PGP-h1 with 3 well-established strains (263K, HY and DY) and literature comparisons with other strains such as Sc237, SHa(Me7), MT-C5, SHa(RML), 139H and Me7-H, also show substantial differences [26],[27]. It is important to note that PMCA amplification of PrP^Sc^ from various strains (of hamsters, mice, human or deer origin) faithfully propagates the strain characteristics [8]–[10],[28],[29]. Thus, the differences observed in the *de novo* generated material are not attributable to PMCA amplification, but to intrinsic differences of this new prion strain. In our direct comparisons the incubation time of PGP-h1 was significantly larger than 263K and HY strains, but much shorter than DY. Clinical signs were also clearly distinguishable from those observed in animals affected by other strains (see Video S1 and Video S2 in supporting online material). The animals inoculated with PGP-h1 were not hyperactive or aggressive, but not lethargic either. They exhibited a social withdrawal and lack of interest for the surroundings, which reflected in a much reduced horizontal and vertical activity and increased extent of inactive time in an open field test. The clinical differences on the PGP-h1 induced disease were likely the result of the severe brain damage produced, including extensive spongiform degeneration, PrP^Sc^ accumulation and brain inflammation. Remarkably, the brain areas targeted by vacuolation damage were significantly different from all other hamster strains studied and included a substantially greater extent of spongiosis in colliculus and a much reduced level of injury in cerebellum and cortex. The biochemical characteristics of PrP^Sc^ were also different in PGP-h1, mostly in terms of the higher quantity of PrP^Sc^ accumulated in the brain and the lower relative resistance to proteolytic degradation than the other hamster strains analyzed. Taken together this data demonstrate that *de novo* generated prions correspond to a novel strain of infectious material, able to generate a new disease in wild type animals. We are currently assessing the infectious properties of some of the other *de novo* generated PrP^Sc^ obtained in this study to examine whether or not new strains and new diseases are produced also in other species.

Our findings provide a model to study the possible origins of sporadic TSEs and a new avenue to investigate the mechanism and factors controlling spontaneous formation of infectious material. For example, using the modified PMCA reaction described in this study we could assess the sequence determinants of the variable propensity of PrP^C^ in distinct species to undergo conversion into the pathological form. We could also study the contribution of other factors in brain to alter the rate of *de novo* generation of PrP^Sc^. Finally, our data provides further support for the prion hypothesis, since misfolded and infectious protein was generated *in vitro*, without the need for addition of pre-existing PrP^Sc^. The fact that the *de novo* generated PrP^Sc^ corresponds to a new strain of infectious material suggest that the diversity of alternative and transmissible foldings that PrP^Sc^ can adopt is much larger than usually thought. This is worrisome, because it raises the possibility that novel and perhaps more aggressive infectious prion foldings may spontaneously originate in diverse species, leading to the emergence of new and unpredictable forms of transmissible diseases.

## Materials and Methods

### Preparation of brain homogenates

Brains from different healthy hamsters, wild type mice, and Tg35 transgenic mice over-expressing human PrP (MM genotype) [30] were extracted after animals were perfused with phosphate-buffered saline (PBS) plus 5 mM EDTA. The human brain used for these studies corresponded to a piece of cortex from a young person (MM phenotype at codon 129) who died from non-neurological disease. The post-mortem delay was 3.5 h. Wild type mice and hamsters were purchased from Jackson laboratory, the human transgenic mice were breaded in our facility and human frozen brain was kindly donated by Dr Pierluigi Gambetti. Ten percent brain homogenates (w/v) were prepared in conversion buffer (PBS containing NaCl 150 mM, 1.0% Triton X-100, 4 mM EDTA and the complete™ cocktail of protease inhibitors from Boehringer Mannheim, Mannheim, Germany).

### PMCA procedure

Aliquots of 100 ul of 10% healthy brain homogenate were loaded onto 0.2-ml PCR tubes. Tubes were positioned on an adaptor placed on the plate holder of a microsonicator (Misonix Model 3000, Farmingdale, NY) and programmed to perform cycles of 30 min incubation at 37°C followed by a 20 s pulse of sonication set at potency of 7. Samples were incubated without shaking immersed in the water of the sonicator bath. Standard PMCA rounds consisted of 144 cycles, whereas extended PMCA consisted of 240 cycles. After each round of cycles, a 10 ul aliquot of the amplified material was diluted into 90 ul of normal brain homogenate and a new round of PMCA cycles was performed. The detailed protocol for PMCA, including reagents, solutions and troubleshooting, has been published elsewhere [31]–[33]. Some of the measures used to minimize cross-contamination were the following: the experiments were done in a laminar flow biosafety cabinet, using disposable materials and aerosol barrier tips. The experimenter was wearing disposable coats, shoe, head and mouth covers and double pair of gloves. All tubes were spin down before opening after any procedure. We avoided spills, clean frequently bench, pipettes and other materials with NaOH, change water and clean sonicator cup with NaOH after each experiment and do not mix prions from different sources in one sonicator. As described in the text, some experiments were carried out entirely in a different laboratory that had never been exposed to prions, using all new reagents, equipment and conducted by an operator who has not been exposed to the prion laboratory.

### Proteinase K degradation assay

The standard procedure to digest PrP^Sc^ consists on subjecting the samples to incubation in the presence of PK (50 µg/ml) during 60 min with shaking at 37°C. The digestion was stopped by adding electrophoresis sample buffer and the protease-resistant PrP was revealed by western blotting, as indicated below. To study the profile of PK sensitivity for *de novo* generated PrP^Sc^, the samples were incubated for 60 min at 37°C with different concentrations of PK ranging from 150 to 1500 µg/ml.

### Western blot

Proteins were fractionated by sodium dodecyl sulphate-polyacrylamide gel electrophoresis (SDS-PAGE), electroblotted onto nitrocellulose membrane and probed with 6D11 antibody at a 1∶5,000 dilution. The immunoreactive bands were visualized by enhanced chemoluminesence assay (Amersham, Piscataway, NJ) using a UVP image analysis system.

### Protein deglycosylation assay

PrP^Sc^ samples were first digested with PK as describe above. After addition of 10% sarkosyl, samples were centrifuged at 100,000×g for 1 h at 4°C, supernatant was discarded and pellet resuspended in 100 µl of glycoprotein denaturing buffer (New England Biolabs, Beverly, MA) and incubated for 10 min at 100°C. Thereafter, 13 µl of 50 mM sodium phosphate, pH 7.5 containing 1% nonidet P-40 and 3 µl of peptide N-glycosidase F (New England Biolabs, Beverly, MA) were added. Samples were incubated overnight at 37°C with shaking and the reaction was stopped by adding electrophoresis buffer and samples analyzed by Western blot as indicated before.

### PrP^Sc^ quantification

To inject the same quantity of PrP^Sc^ from each strain preparation, the samples were compared by Western blotting after PK digestion. To obtain a reliable and robust quantification, we ran several different dilutions of the sample in the same gel, to avoid artifacts due to saturation of the signal or to a too weak signal.

### Infectivity studies

*In vivo* infectivity studies were done in Syrian Golden female hamsters, purchased from Charles River. Animals were 4- to 6-weeks old at the time of inoculation. Anesthesized animals were injected stereotaxically in the right hyppocampus with 2 µl of the sample. The onset of clinical disease was measured by scoring the animals twice a week using the following scale: 1, Normal animal; 2, Mild behavioral abnormalities including tremor of the head and wobbling gait; 3, Moderate behavioral problems including ataxia, head bobbing and irritability; 4, Severe behavioral abnormalities including all of the above plus jerks of the head and body and spontaneous backrolls; 5, Terminal stage of the disease in which the animal lies in the cage and is no longer able to stand up. Animals scoring level 4 were considered sick and sacrificed to avoid excessive pain using exposition to carbonic dioxide. Brains were extracted and analyzed histologically. The right cerebral hemisphere was frozen and stored at −70°C for biochemical studies of PrP^Sc^ and the left hemisphere was used for histology analysis.

### Open field behavioral test

Hamsters at mid stages of the disease (around 1 week after the first clinical signs were observed) were placed in a corner of the field box and its behavior recorded for five minutes. All activity was recorded by a video camera mounted above the open field and scored in real-time. We measured and analyzed total distance, average speed, time spent in various parts of the field, vertical and horizontal activity and inactive time. Testing was carried out in a temperature, noise and light controlled room.

### Histopathological studies

Brain tissue was fixed in 10% formaldehyde solution, embedded in paraffin and cut in sections. Serial sections (6 µm thick) from each block were stained with hematoxylin-eosin, or incubated with the 3F4 monoclonal antibody recognizing PrP or the glial fibrillary acidic protein, using our previously described protocols [3]. Immunoreactions were developed using the peroxidase-antiperoxidase method, following manufacturer's specifications. Antibody specificity was verified by absorption. Samples were visualized with a Zeiss microscope. The vacuolation profile was estimated by considering both number and size of vacuoles. Each analyzed brain area was scored from 0 to 4 according to the extent of vacuolation in slides stained with haematoxilin-eosin and visualized at a 40× magnification. Samples were analyzed blindly by two different persons and the scores represent the average of the two determinations.

### Statistical analysis

The differences in incubation periods, behavioral analysis and histopathoogical profile of brain damage were analyzed by one-way ANOVA, followed by the Dunnett Multiple Comparison post-test to estimate the significance of the differences between PGP-h1 and each of the other hamster prion strains studied. For these studies the data was analyzed using the GraphPad Instat, version 3.05 software.

## Supporting Information

Figure S1 Seeded amplification of diverse healthy brain samples used in this study. To make sure that the healthy brain homogenates used in this study were able to sustain prion replication, different dilutions of vCJD brain homogenates were diluted in 10% brain homogenates of healthy human, MM genotype (Panel A) or humanized transgenic mice, MM genotype (Panel B). All samples were subjected to 144 PMCA cycles. In panel A, frozen (F) and amplified (A) samples are showed next to each other for each dilution, whereas in panels B, frozen samples are showed in the left gels and amplified samples in the right gels. All samples were treated with PK before electrophoresis except when indicated. The capacity of hamster and mouse samples to sustain seeded prion replication is shown in Figure 1C and D of the main text.
(2.71 MB TIF)

Figure S2 Lack of *de novo* formation of PrP^Sc^ in humanized transgenic mice brain homogenate under various conditions. To attempt increasing the rate of spontaneous formation of de novo PrP^Sc^ we subjected the samples of 10% healthy transgenic mice brain homogenates containing human (MM) PrP to various alternative conditions, including: 1. heating the samples at 55°C for 20 min prior to the first round of PMCA; 2. addition of 0.05% digitonin to the conversion buffer; 3. addition of 0.1% SDS to the conversion buffer; 4. changing the pH of the conversion buffer to 5.0; 5. pre-incubation of the brain homogenate under vigorous shaking for 24 h at 37°C. Samples were subjected to serial rounds of 144 PMCA cycles. Thereafter samples were treated with PK and subjected to western blot. The figure shows the results of rounds 3, 6, 9 and 10. No signal was observed in any of the other rounds.
(4.40 MB TIF)

Figure S3 Lack of de novo formation of PrP^Sc^ in hamster brain homogenate under various conditions. To attempt increasing the rate of spontaneous formation of de novo PrP^Sc^ we subjected the samples of 10% healthy hamster brain homogenates to various alternative conditions, including: 1. heating the samples at 55°C for 20 min prior to the first round of PMCA; 2. addition of 0.05% digitonin to the conversion buffer; 3. addition of 0.1% SDS to the conversion buffer; 4. changing the pH of the conversion buffer to 5.0; 5. pre-incubation of the brain homogenate under vigorous shaking for 24 h at 37°C. Samples were subjected to serial rounds of 144 PMCA cycles. Thereafter samples were treated with PK and subjected to western blot. The figure shows the results of rounds 3, 6, 9 and 10. No signal was observed in any of the other rounds.
(3.72 MB TIF)

Video S1 Shows the behavior of hamsters affected by diverse TSE prion strains as well as a control un-infected animal (right box). One animal representative of the disease produced by infection with 263K (center-right box), PGP-h1 (center left box) and DY (left box) is shown. The typical hyperactive behavior of the 263K sick animal, which is constantly moving in the cage, contrast with the complete lethargic posture of the DY sick animal. The PGP-h1 animal has an intermediate behavior, characterized by lack of vertical activity, exploratory interest and substantial inactive time, contrasting with the uninfected animal which is mostly engaged on vertical exploration attempting to escape.
(7.17 MB AVI)

Video S2 Shows a close-up of the behavioral activity of various animals. First, a hamster with clinical signs of the disease produced by PGP-h1 inoculation. Animal exhibit rough coat and clear motor problems including tremor of the head, wobbling gait, head bobbing. It also show a much reduced exploratory behavior and lack of interest for interacting with the object introduced in the cage. The second animal corresponds to a 263K sick hamster exhibiting the typical hyperactivity, motor impairments and aggressiveness. Finally for comparison purposes we show the behavior of a normal healthy hamster of similar age.
(7.69 MB AVI)
