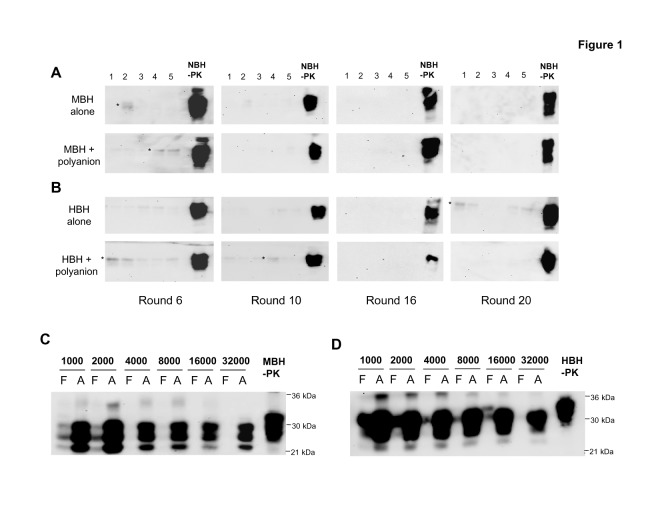# Correction: *De Novo* Generation of Infectious Prions *In Vitro* Produces a New Disease Phenotype

**DOI:** 10.1371/annotation/4b55946a-edb5-4feb-aeb5-2ee160394d17

**Published:** 2013-03-04

**Authors:** Marcelo A. Barria, Abhisek Mukherjee, Dennisse Gonzalez-Romero, Rodrigo Morales, Claudio Soto

A western blot panel in Figure 1 was inadvertently inserted twice to represent both round 16 of MBH + polyanion (Fig. 1A) and round 16 of the HBH + polyanion (Fig. 1B). The authors requested a correction after reviewing the raw data and incorporating the original blot corresponding to HBH + polyanion. The correct blot (new Fig. 1B) does not show any signal besides the migration control of NBH –PK, indicating no spontaneous generation of PrPSc. Therefore, the correction does not change the results or conclusions of this figure or the entire article. The editors have reviewed and agreed to this correction. Please find the corrected Figure 1 here:

**Figure:**