# Supplementary figures and images for: De Novo Generation of Infectious Prions In Vitro Produces a New Disease Phenotype
Source: PLoS Pathog. 2009 May 15;5(5):e1000421. doi: 10.1371/journal.ppat.1000421 (PMC2675078; doi:10.1371/journal.ppat.1000421)

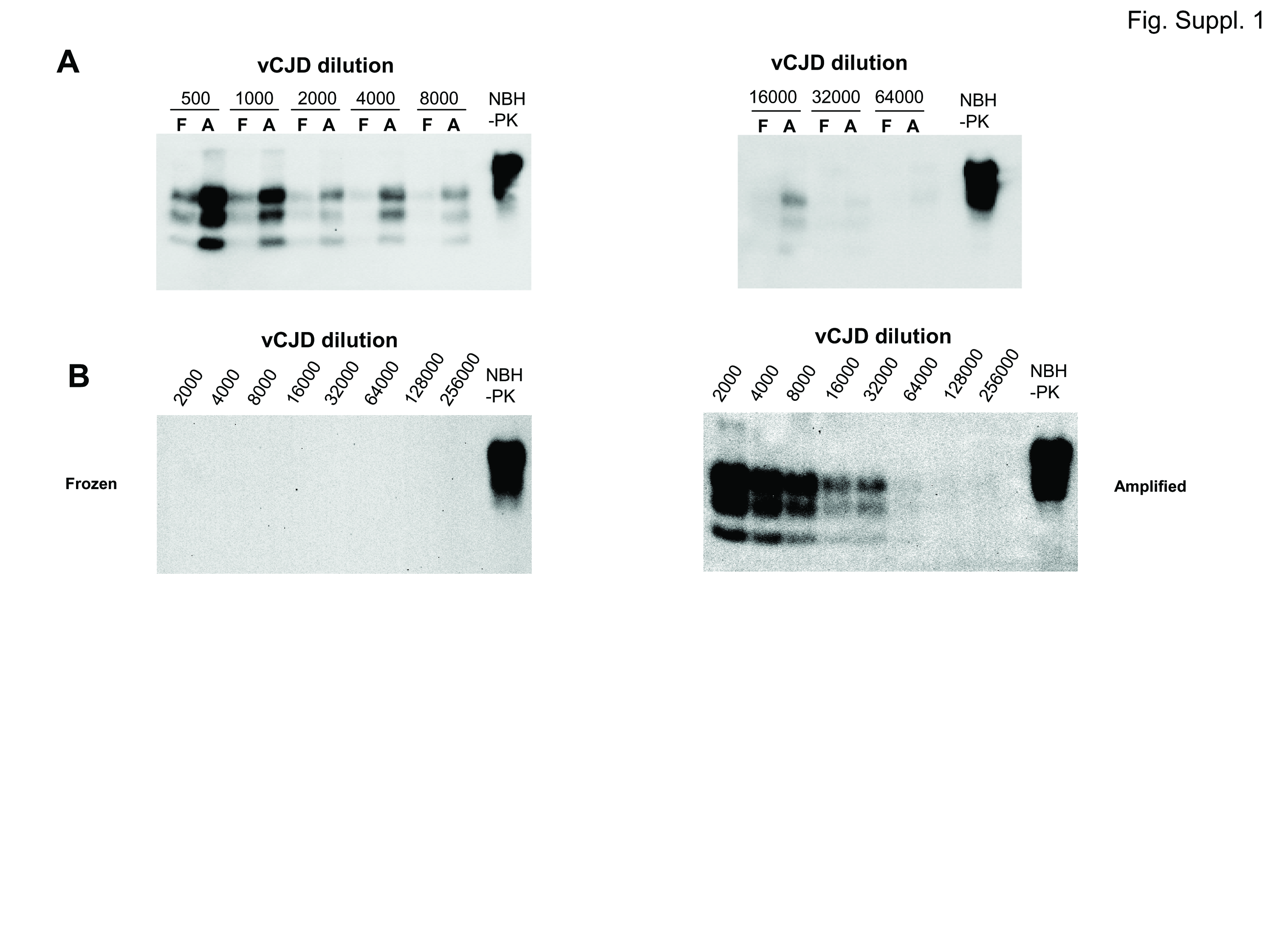

Supplement: Figure S1 — Seeded amplification of diverse healthy brain samples used in this study. To make sure that the healthy brain homogenates used in this study were able to sustain prion replication, different dilutions of vCJD brain homogenates were diluted in 10% brain homogenates of healthy human, MM genotype (Panel A) or humanized transgenic mice, MM genotype (Panel B). All samples were subjected to 144 PMCA cycles. In panel A, frozen (F) and amplified (A) samples are showed next to each other for each dilution, whereas in panels B, frozen samples are showed in the left gels and amplified samples in the right gels. All samples were treated with PK before electrophoresis except when indicated. The capacity of hamster and mouse samples to sustain seeded prion replication is shown in Figure 1C and D of the main text. (2.71 MB TIF) [file ppat.1000421.s001.tif]

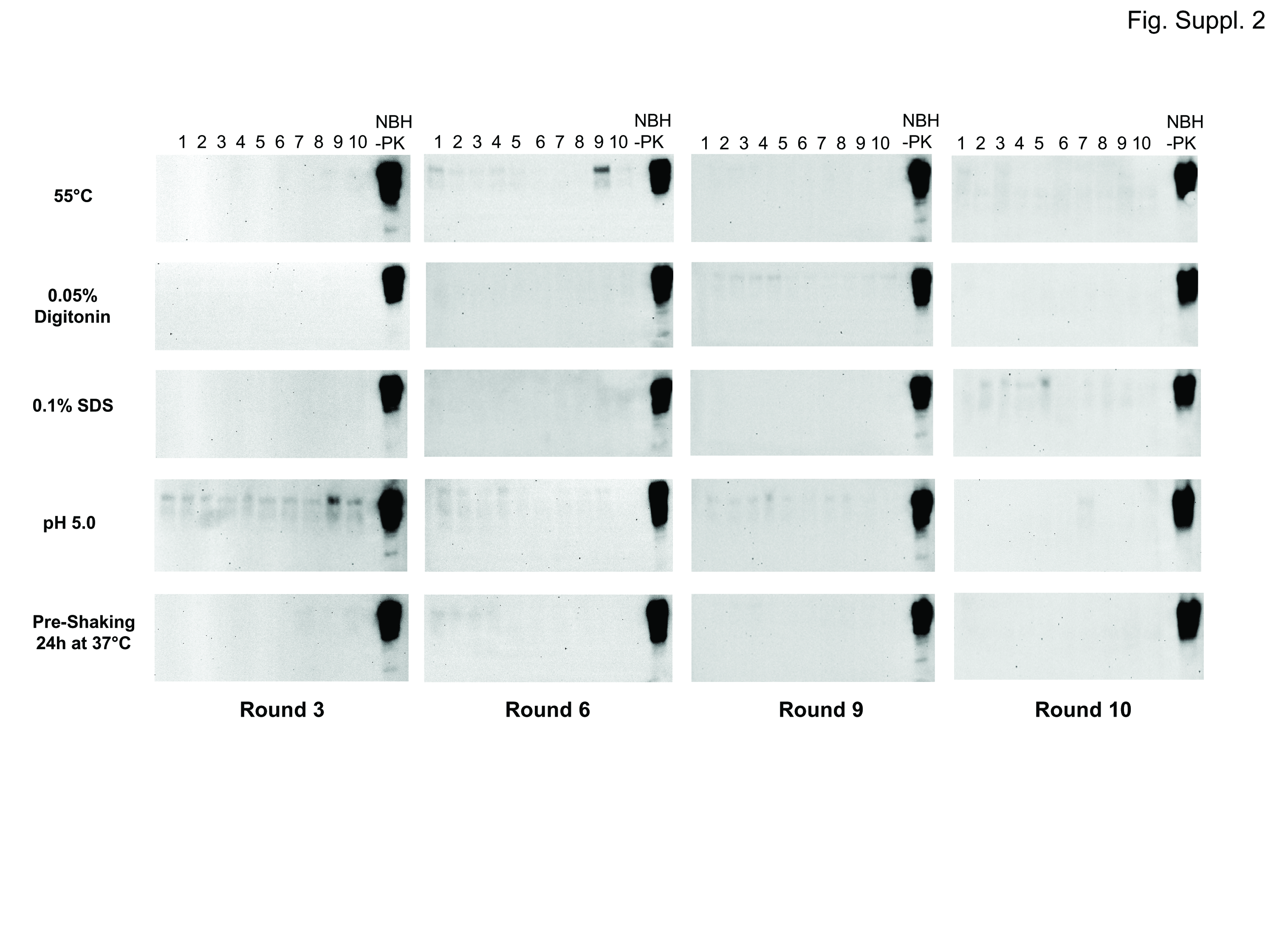

Supplement: Figure S2 — Lack of de novo formation of PrPSc in humanized transgenic mice brain homogenate under various conditions. To attempt increasing the rate of spontaneous formation of de novo PrPSc we subjected the samples of 10% healthy transgenic mice brain homogenates containing human (MM) PrP to various alternative conditions, including: 1. heating the samples at 55°C for 20 min prior to the first round of PMCA; 2. addition of 0.05% digitonin to the conversion buffer; 3. addition of 0.1% SDS to the conversion buffer; 4. changing the pH of the conversion buffer to 5.0; 5. pre-incubation of the brain homogenate under vigorous shaking for 24 h at 37°C. Samples were subjected to serial rounds of 144 PMCA cycles. Thereafter samples were treated with PK and subjected to western blot. The figure shows the results of rounds 3, 6, 9 and 10. No signal was observed in any of the other rounds. (4.40 MB TIF) [file ppat.1000421.s002.tif]

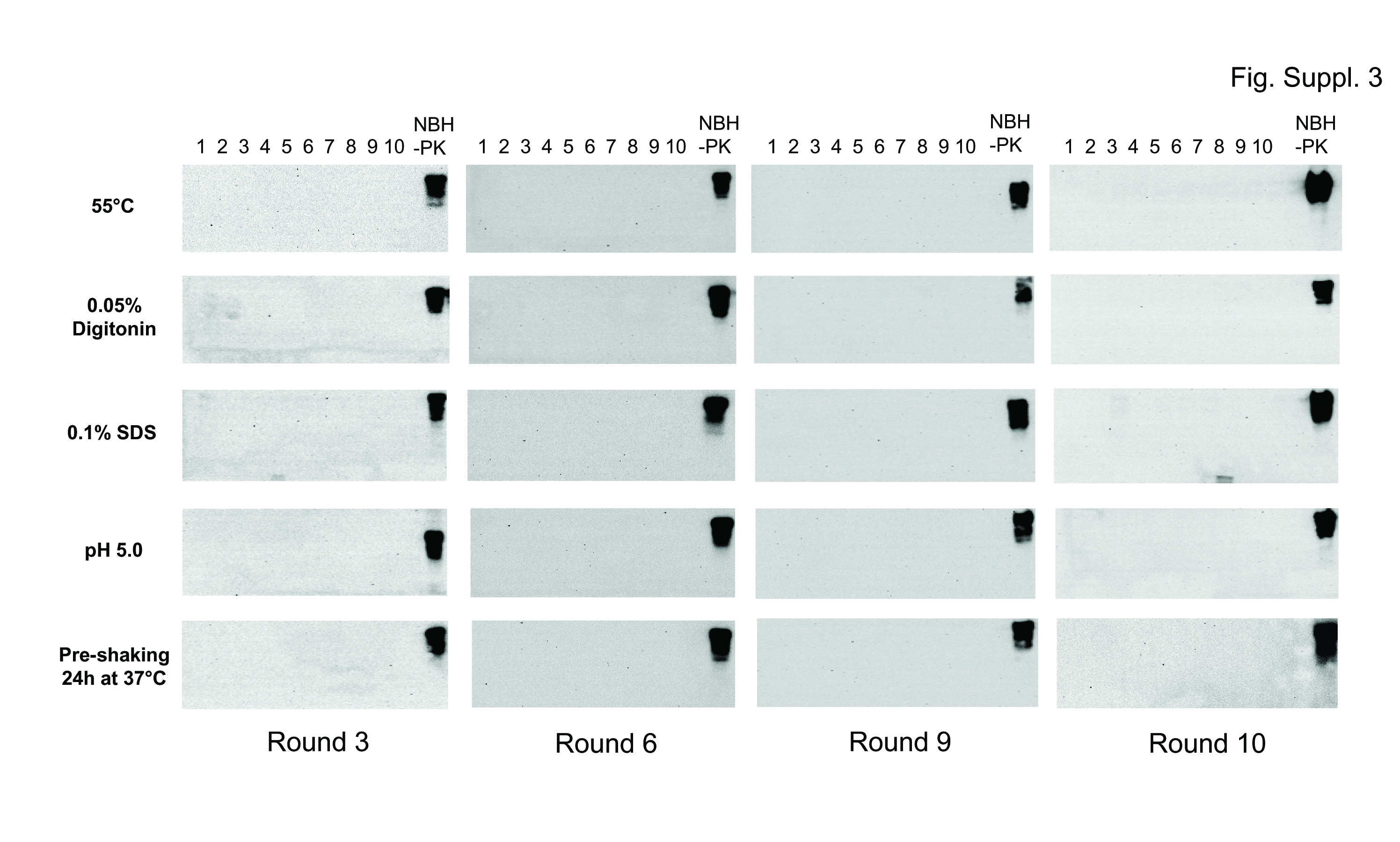

Supplement: Figure S3 — Lack of de novo formation of PrPSc in hamster brain homogenate under various conditions. To attempt increasing the rate of spontaneous formation of de novo PrPSc we subjected the samples of 10% healthy hamster brain homogenates to various alternative conditions, including: 1. heating the samples at 55°C for 20 min prior to the first round of PMCA; 2. addition of 0.05% digitonin to the conversion buffer; 3. addition of 0.1% SDS to the conversion buffer; 4. changing the pH of the conversion buffer to 5.0; 5. pre-incubation of the brain homogenate under vigorous shaking for 24 h at 37°C. Samples were subjected to serial rounds of 144 PMCA cycles. Thereafter samples were treated with PK and subjected to western blot. The figure shows the results of rounds 3, 6, 9 and 10. No signal was observed in any of the other rounds. (3.72 MB TIF) [file ppat.1000421.s003.tif]
